# Spectral Analysis of Dynamic PET Studies: A Review of 20 Years of Method Developments and Applications

**DOI:** 10.1155/2016/7187541

**Published:** 2016-12-05

**Authors:** Mattia Veronese, Gaia Rizzo, Alessandra Bertoldo, Federico E. Turkheimer

**Affiliations:** ^1^Department of Neuroimaging, Institute of Psychiatry, Psychology and Neuroscience, King's College London, London, UK; ^2^Department of Information Engineering, University of Padova, Padova, Italy

## Abstract

In Positron Emission Tomography (PET), spectral analysis (SA) allows the quantification of dynamic data by relating the radioactivity measured by the scanner in time to the underlying physiological processes of the system under investigation. Among the different approaches for the quantification of PET data, SA is based on the linear solution of the Laplace transform inversion whereas the measured arterial and tissue time-activity curves of a radiotracer are used to calculate the input response function of the tissue. In the recent years SA has been used with a large number of PET tracers in brain and nonbrain applications, demonstrating that it is a very flexible and robust method for PET data analysis. Differently from the most common PET quantification approaches that adopt standard nonlinear estimation of compartmental models or some linear simplifications, SA can be applied without defining any specific model configuration and has demonstrated very good sensitivity to the underlying kinetics. This characteristic makes it useful as an investigative tool especially for the analysis of novel PET tracers. The purpose of this work is to offer an overview of SA, to discuss advantages and limitations of the methodology, and to inform about its applications in the PET field.

## 1. Introduction 

Positron Emission Tomography (PET) is a nuclear medicine technique for* in vivo *functional imaging. The system detects pairs of gamma rays emitted at 511 keV after annihilation of a positron-emitting radionuclide (tracer), which is introduced into the body on a biologically active molecule. Thus, depending on the characteristics of the injected tracer, it is possible to derive a large variety of physiological parameters such as blood flow, protein density, enzymatic activity, and metabolism. In order to relate the time-evolving tracer biodistribution to the underlying functional process of interest, the application of mathematical models is necessary. This process is called quantification (from Latin:* quantus* “how much” +* facere* “to make”) and it can be seen as an input/output transformation, where the inputs are represented by the PET measurements acquired during the study (i.e., the concentration of the tracer in the tissues) and the ancillary measurements of radioactive concentration in plasma (e.g., the plasma input function), while the output is represented by a set of parameter estimates describing the tracer kinetics.

For the numerical quantification of PET data several solutions are available [1]; methods range from the calculation of the simple concentration of the tracer in a region of interest up to the complete description of the exchange of the radioactive molecules in the tissue of interest through a compartmental model. The choice of the quantification method is strictly dependent on the purpose of the PET study and on the experimental settings. For example, in PET clinical routine the experimental protocol is generally static, that is, made by a single-frame acquisition at a given time after the tracer administration. This allows easier logistics and maximizes patient throughput. In this setting PET quantification is routinely performed by using standardized uptake value (SUV) [2], a semiquantitative index that is simply computed as the raw image counts normalized by the injected dose and some anthropometric characteristics of the subject (generally the body weight or the body surface area) [3, 4]. SUV is characterized by general applicability but its simplicity may be a limitation if the normalized counts are not associated with the underlying kinetic of interest [5–7].

**Figure 1:**
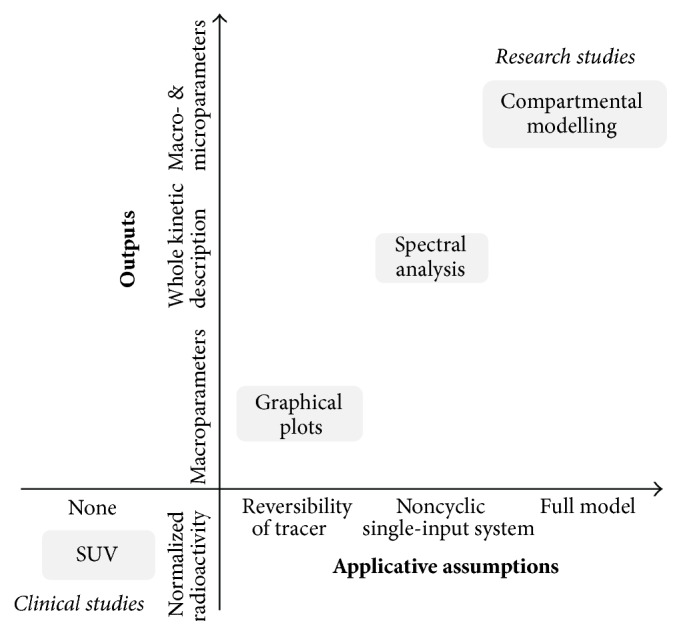
Quantification in Positron Emission Tomography. The figure shows a schematic summary of the major PET quantification methods organized by considering for each approach the information returned as function of the application requirements. Clinical and research PET imaging studies are separately reported. Within the diagram a diagonal distribution of the methodologies is clearly evident, indicating that more information is obtainable only at the cost of more modelling assumptions.

Differently from the clinic, PET experiments in the research setting may require the full dynamic acquisition of multiple volumes over time. This dynamic experimental procedure is necessary to characterize the generally unknown kinetics of the radiotracer in the tissue. Depending on the setting, different analysis approaches are available (Figure 1). The simplest quantification approach for dynamic PET quantification is represented by Logan [8] and Patlak plot [9] graphical methods. Both of them have been widely used for PET quantification, because of their simplicity and the minimal assumptions required for their application. Graphical methods exploit the status of equilibrium that is reached in the system after a certain amount of time from tracer injection, requiring only the information on the reversibility or irreversibility of the tracer kinetics; this leads to a graphical transformation of the data where the parameter of interest is obtained by some form of linear regression. They are easy to implement and, given their linearity, they have been routinely used also for the generation of voxel-by-voxel parametric maps as they are computationally fast and warrant convergence to solution. Nevertheless, graphical plots are affected by several limitations. Both methods allow the estimation of a unique macroparameter (i.e., the tracer net trapping uptake for Patlak and the tracer distribution volume for Logan) but they cannot characterize the whole kinetic profile as they rely on assumptions on the time the equilibrium is reached. In addition they do not account for additional kinetic components such as the vascular signal, for example, the signal generated by the tracer radioactivity in blood cells or plasma or by the radiotracer bound to the vascular walls. Moreover, the linearity of these methods is achieved by transformation of the data that may distort their noise properties and introduce biases, particularly at the large noise levels typical of small resolutions (e.g., small anatomical regions or pixels) [10].

The standard approach for the quantification of dynamic PET studies is represented by compartmental modelling [11, 12]. This approach is based on a first-order differential description of the main physiological processes in which the tracer is involved. Compartmental modelling (CM) requires the full mathematical description of the system under investigation and a complete definition of the model structure, including the type and direction of the tracer exchanges between compartments. CM is the only method that can provide a detailed understanding of the physiological system: hence it is usually adopted as a tool for the investigation of novel tracers only when the compartmental structure describing the underlying system has been characterized. Notably, compartmental models for dynamic PET studies are generally based on a linear time-invariant (LTI) description of the system [13]. Therefore the output of these models can always be represented as the convolution product between the input of the system and its impulse response function (IRF). This condition is always true, irrespective of any nonlinearity between the model output and its parameters.

An alternative approach for the quantification of dynamic PET data is the so-called “spectral analysis” (SA) [14]. The name SA comes from the fact that the IRF of the compartmental models used in PET can be resolved as the analytical sum of exponentials. Hence, the numerical solution of the SA requires the use of the tissue data and the input data to deconvolve the IRF. From the perspective of the theory of signal processing, this equates to finding the poles of the Laplace transform of the IRF. These poles can then be represented as a spectrum of kinetic components that can be attributed to the tracer exchanges between blood and tissues as well as within the tissues. Compared to graphical methods and compartmental modelling approaches, SA represents a good trade-off between the two (Figure 1). For its practical use SA requires the fulfilment of some assumptions about the underlying biological system as it is applicable only to single-input, noncyclic systems [15]; however the large majority of compartmental models used in PET fit these assumptions.

Since its original introduction in 1993, many methodological developments have been proposed to improve the method precision and robustness to the noise. To date, SA along with its filtered versions has been widely used in a large variety of testing conditions, resulting in more than 300 peer-review publications with both preclinical and human data (the literature review included all the papers citing SA original work [14] found in SCOPUS database plus all the papers returned by PUBMED using the research query “Spectral Analysis” AND “PET”; from an initial amount of 393 peer-review papers only 305 effectively referred to SA as quantification method for PET data (date of analysis: 4th August 2016)). This review aims to offer a complete overview of the SA method in PET, from its original formulation up to the subsequent filtered solutions. Applications of SA for kinetic modelling, parametric imaging, tracer model development, and analysis of tissue heterogeneity will be also described, including a summary of the available software for SA implementation.

## 2. Modelling Dynamic PET Data

### 2.1. Compartmental Modelling

CM is the standard approach to quantify tracer kinetic experiments applied to biological systems [16, 17]. At the basis of CM there are (1) the concept of compartment (“*compartment is an amount of material that acts as though it is well-mixed and kinetically homogeneous”* [17]) and (2) the mass-balance equation describing the exchanges of tracer between compartments.

CM can be generally described as follows:

(1) dXtdt=Ap·Xt+Bp·Ut,Yt=Cp·Xt,with Zti=Yti+eti,i=1,2,…,N,

where *p* is the vector of unknown parameters, *X*(*t*) is the matrix of system state variables, *U*(*t*) is the input of the system, *Y*(*t*) is the output of the system, *A*(*p*), *B*(*p*), and *C*(*p*) are the multiplicative matrixes dependent on *p* parameters, *Z*(*t*_*i*_) is the measured output sampled at the time *t*_*i*_, *N* is the number of measures, and *e*(*t*_*i*_) is the measurement error.

Since (1) provides LTI description of the system it can be always be translated into

(2) $$\begin{array}{c} Y\left(\;t\;\right)=U\left(\;t\;\right)⊗IRF\left(\;t\;\right), \end{array}$$

where IRF(*t*) is the impulse response function of the system, that is, the output of the system in case of unitary impulse input.

CM in PET does not make any exception from the general theory, preserving the propriety of LTI models [13]. Specifically, in dynamic PET *U*(*t*) corresponds to the arterial plasma tracer radioactivity (indicated as *C*_*p*_(*t*)), *Y*(*t*) corresponds to the tissue model-predicted radioactivity (indicated as *C*_tiss_(*t*)), *Z*(*t*) corresponds to the PET scan measures, and *X*(*t*) represents the concentration of the radioligand in tissue compartments. For further information about PET compartmental modelling the interested reader is referred to the following works [18, 19].

### 2.2. Spectral Analysis

**Figure 2:**
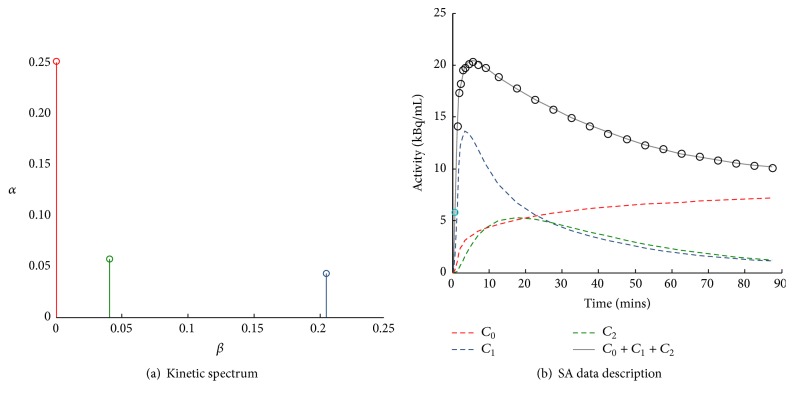
Example of spectral analysis quantification. (a) Representative kinetic spectrum: out of the three spectral components reported, one corresponds to the tracer trapping (red) while the remaining ones refer to two equilibrating components at different frequencies (green and blue). (b) In this example the measured tracer activity (open circles) is described by the sum of the time-activity curves of each individual component of the spectrum (red, green, and blue dashed lines) resulting in the SA data model prediction (grey line). It is important to note that different positions of the components in the spectrum correspond to different shapes of time-activity curves, with the wash-out being slower for low-frequency spectral components and faster for the high-frequency ones.

In SA the tissue tracer activity in a given volume of observation at time *t*, *C*_tiss_(*t*), is modelled as a convolution of the plasma time-activity curve, *C*_*p*_(*t*), with the sum of *M* + 1 distinct decreasing exponential terms as

(3) $$\begin{array}{c} C_{tiss}\left(\;t\;\right)=\overset{M}{\underset{j=0}{\sum }}C_{p}\left(\;t\;\right)⊗\alpha_{j}\cdot e^{-\beta_{j}t}, \end{array}$$

where *α*_*j*_ and *β*_*j*_ (*β*_1_ < *β*_2_ < ⋯<*β*_*M*_) are assumed to be real-valued and nonnegative. This is equivalent to assume IRF(*t*) equal to ∑_*j*=0_^*M*^ *α*_*j*_ *e* ^−*β*_*j*_*t*^. *M* + 1 represents the maximum number of terms to be included in the model and this is, in general, set to a large set (generally between 100 and 1000). The values of *β*_*j*_ are predetermined and fixed in order to cover an appropriate spectral range from the slowest possible event of the tracer in the tissue up to a value appropriate to transient phenomena (e.g., the passage of activity through the tissue vasculature). The values of *α*_*j*_ are estimated from the blood and tissue time-activity curves by a nonnegative least squares (NNLS) procedure. In practice, only a few components with *α*_*j*_ > 0 are detected, originating what is called the kinetic spectrum of the tracer in the tissues (Figure 2). Notably, the nonnegativity constraint of spectral coefficients and components derives from the assumption that SA is modelling a first-order compartmental system with a single arterial input [14].

The estimated spectral components assume different meanings depending on the position of the beta grid in which they are located. For example, the terms for lim *β*_*j*_ → *∞* (i.e., components with *β*_*j*_ very large) become proportional to *C*_*p*_(*t*) and can be considered as “high-frequency” components. In the same way the corresponding term with *β*_*j*_ = 0, or near zero, becomes proportional to ∫*C*_*p*_(*t*) and can be viewed as the “low-frequency” component, that is, accounting for slower kinetics and limiting to its irreversible trapping in the tissue. Components with intermediate values of *β*_*j*_ (“equilibrating components”) reflect tissue compartments that exchange material directly or indirectly with the plasma with their number corresponding to the number of identifiable tissue compartments within the volume of interest. In light of these particular features, it is very common to define the SA model equation explicitly showing trapping in the following way:

(4) $$\begin{array}{c} C_{tiss}\left(\;t\;\right)=\alpha_{0}\cdot \overset{t}{\underset{0}{\int }}C_{p}\left(\;\tau \;\right)d\tau +\overset{M}{\underset{j=1}{\sum }}C_{p}\left(\;t\;\right)⊗\alpha_{j}\cdot e^{-\beta_{j}t}, \end{array}$$

where *β*_*j*_ > 0, *j* = 1,2,…, *M*.

### 2.3. Derivation of the Major Parameters of Interest

The main purpose of SA application to dynamic PET data is the quantitative characterization of the tracer kinetics within the target tissues. This is possible by linking the estimated spectral components, that is, the estimated *α*_*j*_ and *β*_*j*_, with macroparameters of interest that do not depend on a specific model representation. As demonstrated by Gunn and colleagues [13], when a particular system meets the conditions to be modelled with spectral analysis (the SA applicability is limited to those systems in which the state transition matrix is negative semidefinite; this condition is met by all single-input/single-output noncyclic systems (for full mathematical derivation please refer to Schmidt [15])) the unique identifiability of some macroparameters of interest is guaranteed. These parameters are the influx rate constant (*K*_1_, mL/cm^3^/min), the net uptake of the tracer in the tissues (*K*_*i*_, mL/cm^3^/min), and the volume of distribution (*V*_*T*_, mL/cm^3^) (the measurement units here reported for *K*_*i*_, *K*_1_, and *V*_*T*_ follow the guidelines of PET modeller consensus [20]). The last two elements cannot be estimated simultaneously, because they depend on the irreversibility or reversibility of the tracer kinetic, respectively. In addition to these parameters of interest, the estimated spectrum provides information also on the number of the system components necessary to describe the data and on their type (i.e., reversible/equilibrating or irreversible). This characteristic of SA can be very useful when new PET tracers are investigated for the first time. The relationship between the parameter values and estimated spectrum is as follows. The transport of tracer from plasma to tissue, *K*_1_, coincides with the sum of components' amplitudes; that is,

(5) $$\begin{array}{c} K_{1}=\overset{M}{\underset{j=0}{\sum }}\alpha_{j}. \end{array}$$

In case of irreversible tracers, *K*_*i*_ can be derived by the limit of the SA IRF(*t*) for *t* → *∞*, which is also equal to the amplitude of the estimated component corresponding to *β*_*j*_ = 0:

(6) $$\begin{array}{c} K_{i}=\underset{t\to \infty }{lim}\;IRF\left(\;t\;\right)=\underset{t\to \infty }{lim}\;\overset{M}{\underset{j=0}{\sum }}\left[\;\alpha_{j}\cdot e^{-\beta_{j}t}\;\right]=\alpha_{0}. \end{array}$$

In case of reversible tracers, instead, *V*_*T*_ can be computed from the integral of IRF(*t*) as

(7) $$\begin{array}{c} V_{T}=\overset{\infty }{\underset{0}{\int }}IRF\left(\;\tau \;\right)d\tau =\overset{M}{\underset{j=1}{\sum }}\frac{\alpha_{j}}{\beta_{j}}. \end{array}$$

In addition to these parameters, if the measurement equation for the total radioactivity measured by the PET scanner takes into account the tracer contribution in both blood and tissues, it is also possible to derive the blood volume (*V*_*b*_, unitless). Generally, this corresponds to the case in which

(8) $$\begin{array}{c} C_{measured}\left(\;t\;\right)=\left(\;1-V_{b}\;\right)\cdot C_{tiss}\left(\;t\;\right)+V_{b}\cdot C_{b}\left(\;t\;\right), \end{array}$$

where *C*_measured_(*t*) represents the total activity measured by the scanner within a specified volume of observation, *C*_tiss_(*t*) represents the tissue kinetic activity, and *C*_*b*_(*t*) represents the whole blood tracer activity.

**Figure 3:**
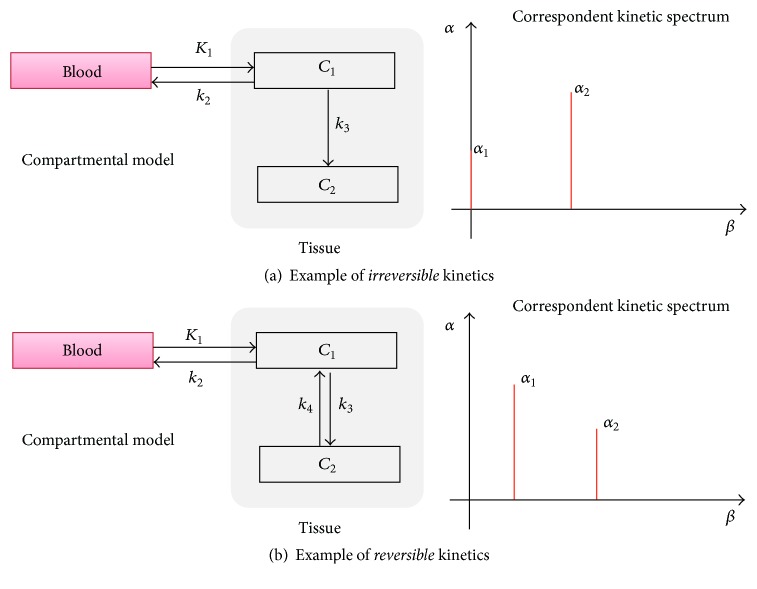
Compartmental models and correspondent kinetic spectra. Two-tissue model with trapping (a) and standard two-tissue compartmental model (b) are reported as example of models for tracers with irreversible and reversible kinetics, respectively.

From the analysis of the SA estimated spectrum, however, it is not possible to compute the microparameters of the system, unless a full characterization of the compartmental model describing the system is known in advance. In fact, from the indistinguishability theorem* “any two plasma input models, either reversible or irreversible, with a total of N tissue compartments are indistinguishable”* one can discern that different compartmental arrangements may return the same kinetic spectrum [13]. The lack of a unique bidirectional relationship between SA spectra and compartmental models prevents the identification of a unique compartmental model given a specific spectrum and thus a unique set of system microparameters. Note however that this limitation pertains to CM* per se* and not to SA. On the contrary, given a compartmental model which fulfils the SA requirements (i.e., noncyclic and with single arterial input), there is a fully described relationship between the spectral components and the model configuration (Figure 3). In summary, SA returns multiple kinetic parameters (not only *K*_*i*_ or *V*_*T*_), accounts for all tissue components including the vascular tracer presence, and returns the model fit to the data.

## 3. Method Implementation

### 3.1. Exponential Spectral Analysis (ESA)

In the first publication on SA, Cunningham and Jones proposed a linear estimator to solve (1), defining what is also known as Exponential Spectral Analysis (ESA, Table 1) [14]. The idea is to fix the possible values of *β*_*j*_ covering a biological plausible spectral range of *M* + 1 elements, making in this way the problem linear in the parameters. For the studies involving short lived positron-emitting isotopes this range needs to extend to the slowest possible event of the tracer in the tissue up to a value appropriate to transient phenomena (e.g., the passage of activity through the tissue vasculature) [15]. In order to obtain a sparse and unique solution, that is, only a few components with *α*_*j*_ > 0 detected, the values of *α*_*j*_ are estimated from the blood and tissue time-activity curves by NNLS procedure, originating what is called the kinetic spectrum of the tracer in the tissues.

The factors determining which components are identified are mainly the distribution of betas and the weighting approach implemented in the estimator [21]. The grid of *β*_*j*_ is generally defined as a logarithmic distribution [21, 22] (see also [23] for some interesting considerations about the spacing of the grid). As general rule (derived from the control theory for linear systems) the lower limit of the distribution is defined as *β*_1_ = 1/(3 · *T*_end_), where *T*_end_ is the end time of PET study, and the upper limit is defined as *β*_*M*_ = 3/*T*_in_, where *T*_in_ is the duration of the first PET frame of the study. However, due to the discrete nature of the component grid, the optimal ${\hat{\beta }}_{j}$ solutions leading to the best data fitting might not necessarily be available. When an adequate approximation of the optimal ${\hat{\beta }}_{j}$ is not on the grid, the algorithm chooses instead two consecutive values of betas that bracket the optimal value. This effect is called “doubling” and can be solved by replacing each estimated pair of components with the average of the two components weighted by their spectral coefficients [24].

Consistently with other PET quantification methods, SA weights are defined as the inverse of the variance of the PET measurement error, which is assumed to be additive and uncorrelated from a Gaussian distribution with zero mean.

The spectral analysis model has general applicability; however its functional components *C*_*p*_(*t*)⊗^(−*β*_*j*_*t*)^ represent an overcomplete basis of the space of interest (in functional analysis a common way to represent real-valued signals is with a linear superposition of basis functions; when the number of basis vectors is greater than the dimensionality of the signals to be represented, the basis is said to be overcomplete; under an overcomplete basis, the decomposition of a signal is not unique [25]) [26]. This results in two main problems: first of all the error properties of the estimates as well as the influence of the error on the estimated components are difficult to estimate and control [27]; secondly to identify a unique and sparse solution the coefficients *α*_*j*_ must be constrained to be positive. All the compartmental models with plasma or blood input functions and noncyclic structures result in positive *α*_*j*_ values [15], allowing the applicability of ESA to most of the kinetic models used with PET; however this nonnegative constrain does not apply necessarily when the input used is not plasma but, for example, the TAC of a reference region with no or negligible amount of tracer specific binding. When a reference region is present in the PET field of view, it can be used as proxy of arterial input function to represent tracer delivery to the tissues [28]. Unfortunately, IRF(*t*) for reference region models may result in both positive and negative *α*_*j*_ and therefore SA is no longer applicable [13]. Thus alternative regularization strategies need to be used to identify a unique solution. Examples of these approaches are represented by the Monte Carlo optimization proposed by Maltz [29] or by the rank-shaping regularization by Turkheimer et al. [30].

### 3.2. Filtered SA Solutions

ESA is well known to be sensitive to the noise in the data [21, 23, 27, 31], with the bias being highly dependent on the level of noise present, making its application in general preferable for high SNR data like in region level analysis. To improve its robustness to the noise, over the years different alternatives have been proposed.

The first attempt to regularize the solution of (1) was done by including penalty functions on the NNLS algorithm [14]. However, the definition of an adequate penalty function to use in practice has proved to be difficult. This concept was further developed by Gunn and colleagues in 2002 and it originated what are now called basis pursuit methods [31, 32]. In 1994, Turkheimer and colleagues proposed a high-pass filter for equilibrating components, with the aim of improving estimates of *α*_0_ and thus determining a more accurate and precise estimate of regional cerebral metabolic rate for glucose in PET studies [21]. Even though the use of this filter has been shown to produce better estimates of *α*_0_ compared to the standard ESA, it represents an incomplete solution. In particular this method does not account for the noise-derived components (also known as “phantom” components) at intermediate and high frequency [24], which, as well as the low-frequency components, contribute to the description of tissue tracer kinetics.

In 1993 and 1997, Takodoro et al. [33, 34] proposed using the SA impulse response function as an alternative quantitative metric; IRF(*t*) is indeed more robust to the noise in the data but its use is limited by the lack of a clear relationship with the underlying biology. In 1998 two different alternatives were proposed to account for SA noise properties. First of all, a bootstrap approach based on residual resampling was implemented to simulate the effect of the noise on the SA spectrum and to correct for possible bias [27]. The resulting bootstrapped spectrum was shown to be a good estimator of the average spectrum that would have been obtained if repeated samples of the measured data were available. However, because the procedure is computationally intensive, it may be inapplicable in the case of very large datasets such as in voxel-wise PET analysis. In the same year, a new procedure for the suppression of noise artefacts on the estimated SA spectrum was proposed by Cunningham and colleagues [23]: standard statistical tests and information criteria were applied to subselect the true estimated components from a given SA spectrum, removing those related to the noise in the data.

Parallel to the developing of new SA improved versions, some efforts were done to denoise the PET images, exploiting the idea that quantification can be improved by increasing the SNR of the analysed data. Several solutions have been proposed in literature, but, specific to SA methodology, the most significant attempts were represented by the use of* wavelets* [35, 36] and by the* functional smoothing approach* [37]. Despite their great potential, both of these approaches have had limited impact in the PET modelling community.

At the present time, the most successful filtered versions of the SA method are represented by rank shaping (RS) [30] and spectral analysis with iterative filtering (SAIF) [24], available for the parameter estimation of low SNR data (Table 1). RS is a Bayesian development of standard SA, optimized for the estimation of the volume of distribution for reversible tracers, which is based on the same principles of SA but without requiring the nonnegative constraint of the spectral components. To overcome the high sensitivity of the unconstrained SA to the noise in the data, RS implements a Kalman filter of the estimated kinetic spectrum providing reliable estimates of *V*_*T*_ (for the derivation of this method, the interested reader is referred to the original reference by Turkheimer and colleagues [30]). Unlike other SA-based methods, RS can be applied with an arterial input function as well as with a reference region. As for ESA, RS requires the definition of the grid of components. In addition, RS requires the SNR interval bound values of the Kalman filter to be set, which is based on a signal-to-noise (SNR) estimate of the data to be processed.

**Figure 4:**
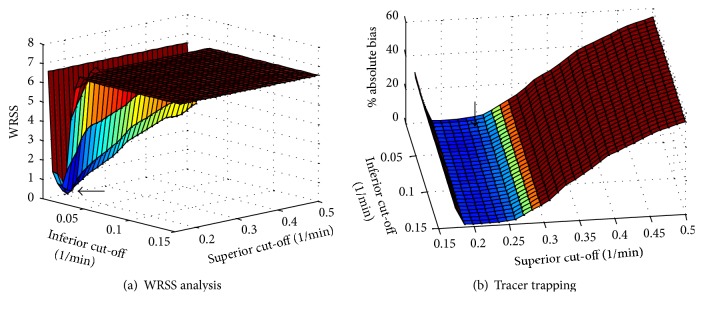
Sensitivity of SA filter methods to filter setting. The figure shows the performance of SAIF when different passband bounds are used to fit a particular dataset, corresponding to a simulated spectrum with two equilibrating components and one trapping. Both fit performance (indicated by weighted residual sum of squares, WRSS) and parameter estimates bias (tracer trapping, *K*_*i*_) can be heavily affected by filter setting. It is important to note that the filter bounds returning the lowest bias do not match those returning the best data fit (black arrows). This indicates that the filter setting cannot be determined only on the basis of data fit quality.

SAIF is the complementary approach for low SNR PET data quantification of irreversible tracers optimized for the estimation of *K*_*i*_ and *K*_1_ [24]. SAIF implements a passband filter [*β*_*L*_,  *β*_*U*_] to separate trapping and blood components from the equilibrating ones: all *β*_*j*_ estimates within the filter passband are preserved, while those outside the filter are removed from the spectrum. Because the removal of these components affects the blood and trapping estimates, both estimation and filtering steps are repeated until stabilization of the weighted residual sum of squares (for the derivation of the method the interested reader is refer to the original reference by Veronese and colleagues [24]). As ESA, in addition to the estimated macroparameters *K*_*i*_, *V*_*b*_ and *K*_1_, SAIF returns also the model fit and the kinetic spectrum. As RS, the method can be applied both at the region and at the voxel level and it requires the definition of the grid of components as well as the filter setting. This last choice is not trivial, since the method performances are heavily affected by its definition (Figure 4).

## 4. Applications

### 4.1. Parametric Imaging

Parametric mapping with PET is possible only when the quantification is performed for each voxel of the image. However, due to the low SNR of the voxel kinetics and the very high number of voxels to be analysed, PET parametric imaging can be very challenging. Voxel-wise analysis requires large computational time and convergence to a unique solution is not always guaranteed for all the voxels.

**Figure 5:**
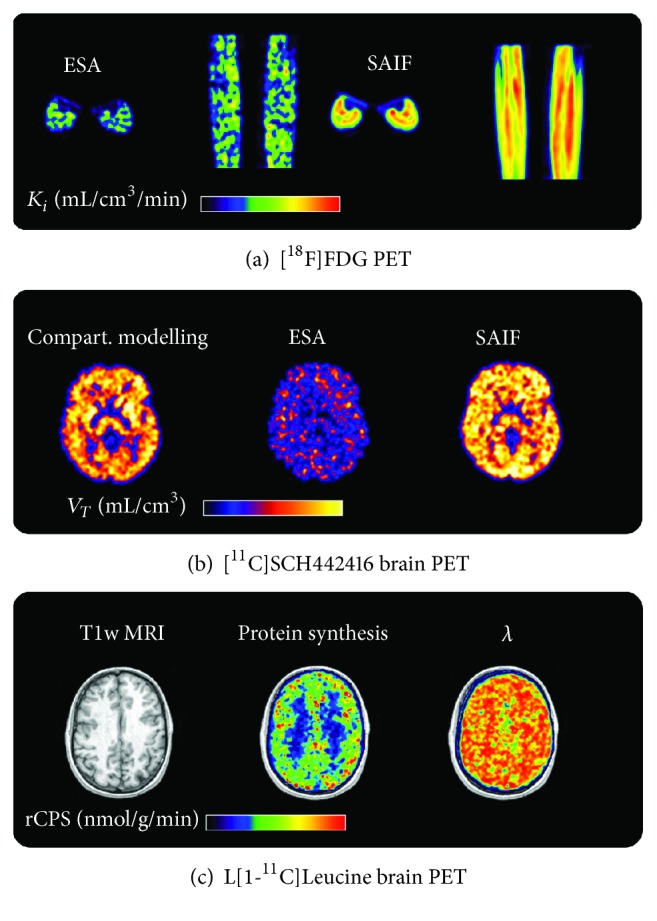
Parametric imaging with SA. (a) *K*_*i*_ parametric maps obtained with ESA and SAIF applied to [^18^F]FDG PET data. Results refer to the midcalf area in one representative subject, reported in both axial and coronal views. (b) *V*_*T*_ parametric maps obtained with ESA and a modified version of SAIF applied to [^11^C]SCH442416 brain PET data. Results refer to a representative transaxial slice of a healthy volunteer. *V*_*T*_ map obtained from compartmental quantification is also reported for comparative purposes. (c) Rate of cerebral protein synthesis (rCPS) parametric map obtained with SAIF applied to L[1-^11^C]Leucine brain PET data. Results refer to a representative transaxial slice of a healthy volunteer. T1-weighted structural MRI and the fraction of blood-derived leucine (*λ*) are also reported for comparative purposes.

Among the different alternatives that have been introduced in literature (for an overview see [38–41]), spectral-based methods have shown to be efficient and precise solutions for parametric imaging both in brain and in nonbrain tissue (Figure 5). In general better performance can be achieved with SA filtered solutions (RS or SAIF), which have shown superior robustness to the measurement noise typical of voxel-wise analysis [42–44]. Notably, SA model can be embedded in the PET image reconstruction allowing tracer kinetic quantification directly from the PET sinogram [45, 46].

### 4.2. Model Development

In addition to tissue kinetic quantification, SA has been used as an investigative tool to model newly introduced PET tracers or when biological systems have been explored for the first time [47–50]. In this context, SA spectra offer the possibility of determining the number of compartments present in a system as well as their types, distinguishing between equilibrating components and irreversible tracer uptake. However, it remains impossible to determine an unequivocal correspondence between the spectrum and its equivalent model (*indistinguishability theorem*, [13]). Thus, for a particular estimated spectrum it is only possible to associate a class of equivalent compartmental representations which have in common the same number of compartments [1]. Then, using the physiological knowledge of the system, it is possible to choose from these alternatives the optimal compartmental configuration for the description of the kinetics of the tracer under study. This procedure is theoretically always applicable but may not be advisable for real practice. Often the presence of noise in the data (especially for data with low SNR) leads to a biased number of SA estimated components (generally overestimated) and hence to an erroneous class of model configurations [49].

A better approach to follow is simply to estimate the number of exponentials necessary to fit the data by defining a set of model alternatives of increasing order, identifying each of them and selecting the one that best describes the data. The standard model parsimony criteria techniques (Akaike or Bayesian information criterion) can be used as decision indexes. This approach, also called nonlinear spectral analysis (NLSA, Table 1), offers several advantages compared to standard SA [49]: (1) the precision of both *α* and *β* estimates is provided. This information can be combined with the parsimony indexes for model selection; (2) the estimation of *β* within a prefixed compartmental structure avoids the problem of the extra components as in the standard SA.

NLSA has been used in different contexts, including brain, heart, and lung PET imaging [49, 51]. Notably, given the general applicability of NLSA, its use has been extended to other fields as magnetic resonance imaging [52, 53] or for characterizing the catabolic plasma concentration decay in dialysis [54].

### 4.3. Tissue Kinetic Heterogeneity Measurement

**Figure 6:**
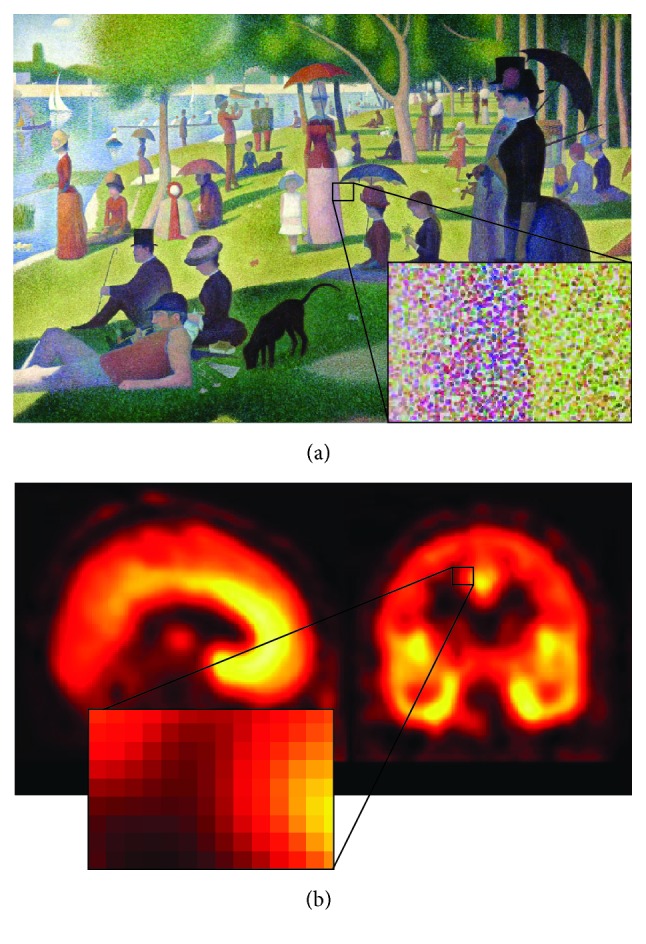
Tissue kinetic heterogeneity in PET domain. (a)* The Sunday Afternoon on the Island of La Grande Jatte* (1886). This painting from George Seurat is one of the most famous examples of pointillism, but it can be also used as a schematic representation of kinetic heterogeneity concept. From a visual analysis, it appears clear that the whole picture is the result of several graphics elements which all contribute to the final effect of the painting. If we zoom in a particular area of the painting we can now visualize the single constitutive elements of colour. It is following this hierarchical organization, based on the combination of small points of colour, that the famous painting is obtained. Unfortunately the same concept cannot be applied directly to PET imaging (b). Due to the finite spatial resolution of the modality, it is not possible to zoom in until the constitutive elements of the tissues are individually reported. The best analysis is limited at the voxel level, where each voxel represents the mean activity measured in the tissues within its volume. This assumption can be acceptable only when the tissue within the voxel volume is a homogeneous mix and therefore identifiable through the mean operator. On the contrary, when the voxel contains a mixture of different tissues, like when it is located at the border between grey and white matter, the heterogeneity of the tissues must be taken into account for a correct quantification.

When a physiological process is investigated* in vivo*, it is reasonable to expect a variability of the response. This variability is not* a priori *predictable as it is the result of a combination of different uncontrolled factors. Intuitively, the higher the complexity of a system of interest, the higher the variability observed. This concept is based on the idea that heterogeneous systems are characterized by higher variability compared to correspondent homogeneous ones, as similar elements tend to have similar behaviour. Theoretically, it is possible to assume that heterogeneity becomes negligible only when the system can be broken up in homogeneous subsets of elements. This condition is very difficult to be reached with any biomedical imaging modality (including PET) because of the limited spatial resolution (Figure 6). Given that the intrinsic resolution of standard scanners is ~5 mm, it is easy to see that a resolution element will contain at least portions of different tissues; in brain, for example, cortical thickness never exceeds 3 mm [55]; hence every voxel will contain tissue elements from grey and white matter that are characterized by different perfusion and metabolic rates.

**Figure 7:**
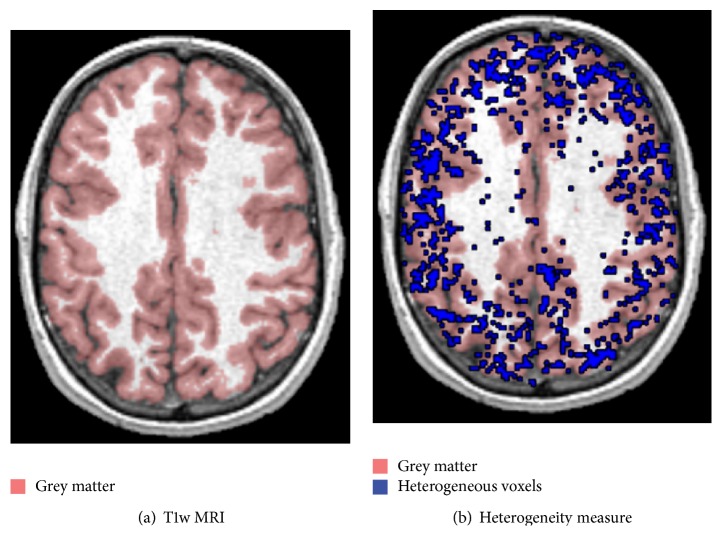
Kinetic inhomogeneity analysis in L-[1-^11^C]Leucine. The figure shows T1-weighted MRI image (a) and the same anatomical picture fused with the spatial distribution of voxels estimated as heterogeneous (b). The blue points correspond to those voxels where SAIF algorithm detects the presence of at least two equilibrating components from the voxel time-activity curves analysis. The red area, instead, highlights the grey matter tissue as well as its border with white matter.

Application of kinetic models designed for homogeneous tissues to heterogeneous tissues has been shown to lead to errors in estimated rates of cerebral blood flow and glucose metabolism, as well as to errors in estimates of receptor binding parameters [56–58]. Because SA does not require the number of compartments to be fixed* a priori*, it applies to heterogeneous as well as the homogeneous tissues without any additional assumptions. In fact, whenever a homogeneous tissue model can be appropriately analysed with SA, its heterogeneous counterpart can be modelled as well without introducing any bias [26]. Moreover, by evaluating the number of estimated kinetic spectra, SA can identify the number of subregions composing the system of interest, returning a measure of heterogeneity degree of a particular volume of interest (Figure 7). This information is linked to the complexity of the tissues and can provide useful insight when applied to pathology [51, 59, 60].

## 5. Clinical and Preclinical Use

The first application of the SA model [14] (as ESA) was done with brain PET datasets, specifically for the evaluation of cerebral blood flow and cerebral glucose utilization and for opiate receptor ligand binding. H_2_^15^O, [^18^F]FDG, and [^11^C]DPN dynamic PET data were considered for this purpose. After these initial applications, the SA model has been widely used in a large variety of testing conditions, both in preclinical and in clinical studies, considering different tracers and receptor systems, both in its original and in filtered formulations.

ESA has been applied to animal (i.e., mice, rats, rabbits, and nonhuman primates) [61–64] as well as to clinical data. Most of its applications are related to the investigation of brain tissues, counting more than 100 different studies. Some meaningful examples are the applications with [^11^C]diprenorphine [65], [^11^C]PIB [66], [^11^C]flumazenil [67], [^11^C]RO15-4513 [50], [^11^C]befloxatone [68], and [^18^F]FIMX [69] in many different conditions including psychiatric conditions, neurodegeneration, and epilepsy. The flexibility of the algorithm allowed the extension of the method also to the analysis of nonbrain tissues, such as heart [70, 71], skeletal leg muscle [47, 72, 73], bone [74, 75], liver [76, 77], and kidney [78]. Of particular importance are the applications in the oncology field to breast, lung, and gastrointestinal cancer, to identify abnormal tissue kinetics and to characterize the pharmacodynamics proprieties of anticancer drugs [79–84].

RS has been shown to be a precise and accurate quantification method [30, 65, 85–88], demonstrating that it is a reliable tool for parametric imaging, even in high-noise conditions [30]. It is of particular interest since it allows model-free quantification using reference region; however it is always important to check the validity of such reference to avoid the introduction of systematic bias [89].

SAIF was originally developed for quantifying rates of cerebral protein synthesis with L-[1-^11^C]Leucine [24, 42], but it has also been applied also to measure cancer proliferation and lung inflammation after injury [51, 59]. In all cases, it has been shown to be a robust and accurate estimator for region level analysis and parametric mapping of PET tracers with irreversible uptake.

## 6. Software Support

Despite the great potential offered by standard and filtered spectral analysis algorithms, the use of this methodology in the PET community is limited to few research centres. The main reason for this poor diffusion coincides with the lack of a unified and complete software environment for SA application. Generally, SA routines are realized with in-house code (e.g., Clickfit, from Imperial College, or MICK from Manchester University) which are rigidly linked to specific data format. This narrows the application of SA only to a restricted number of people that represent a very small subset of researchers working with PET who could take advantage of SA usage.

In the last few years, SA methods have been implemented in software packages available for a comprehensive elaboration of dynamic PET data such as SAKE (Spectral Analysis Kinetic Estimation, http://bio.dei.unipd.it/sake/cgi-bin/index.cgi, [44]) and PMOD (since Release 3.4 in 2012, https://www.pmod.com/web/). Among these software programmes, SAKE is the only one that implements also the filtered RS and SAIF version, working both for brain and for nonbrain tissues. All the software programs listed work through a Graphical User Interface (GUI); thus no programming knowledge is required, in order to facilitate its use also by nonexpert IT users.

## 7. Discussion

### 7.1. Advantages of SA Quantification Methods

The main strength of SA methods is related to the flexibility of the implemented model: due to its additive formulation, SA can be applied to reversible/irreversible kinetics, single compartment or multicompartment models, and homogeneous as well as heterogeneous systems. This characteristic makes SA adaptable to different tracers and different physiological systems without any* a priori* assumption concerning tracer exchanges within or across the tissues of interest.

SA represents a complete quantification tool for dynamic PET analysis returning a valuable set of macroparameters estimates (like trapping or tracer transport) and a full model description of the entire data time-course, without requiring a fixed model structure. SA can be also used for model development for the identification of the number and type of compartments necessary to describe a given system. In presence of heterogeneous systems, SA can be used as robust tool to investigate the spatial distribution of tissue complexity.

Within its applicability domain, SA has demonstrated to be easily modifiable to the specific characteristics of the system under study as in the case of tracers such as [^11^C]PK11195, [^11^C]SCH442416, and [^11^C]PBR28 [85, 90, 91] or with the double input SA [92]. In the first cases the structure of SA model was changed to account for the endothelial binding of the tracers; in the second case, instead, the SA model incorporated the presence of metabolites within the tissues under study. In both situations the structure of SA model was enriched with additional components whose amplitudes were then estimated from the data as the others already present in its functional basis.

### 7.2. Limitations of SA-Based Quantification Methods

For its definition SA requires the assessment of the tracer concentration over time in arterial plasma. This information is obtained by arterial blood sampling, which could be performed manually by an operator or automatically by an appropriate device. Arterial line, however, represents a risk of the patient (e.g., infections or strokes) and a risk for the personnel (e.g., risk of handling the blood of the patient or exposure to extra radiation), characteristics that limit its use in common practice. With the development of noninvasive input function techniques, some alternatives to the arterial sampling are present including image-derived input functions [93], population-based input functions [94], or venous input function [95]. However their use with SA is still limited as the estimated spectrum is strictly dependent on the shape of the arterial input, and small variations on its time-course can lead to high biased estimates, both for micro- and for macroparameters [96]. Moreover, the application of noninvasive methods results tends to increase estimate uncertainty, which becomes a problem when groups of subjects have to be statistically compared. At the moment, arterial blood sampling remains the standard for SA-based method applications.

SA method is characterized by well-defined applicability limits, which derive from the nonnegative constraint used to estimate the coefficients of its basis functions. The main drawback of this assumption is the restriction of SA applicability only to models with a unique input function and without cycling connection [15]. Most of the models used in PET met these conditions, but unfortunately reference regions models do not belong to this category. It is important to stress the concept that SA cannot be applied to reference regions, not because reference region models are inadequate to be described by sum of convolution terms but because it is not possible to estimate their kinetic spectra imposing the nonnegative constraint of the spectral coefficients.

Filtered versions are characterized by different (generally smaller) application domains. SAIF and RS, for example, require the irreversibility/reversibility of the tracer kinetics, respectively. This characteristic of the tracer has to be verified prior to their applications. RS, however, can be applied to reference region models for the distribution volume estimation. The larger applicability domain of RS is offered at the cost of a decreased number of outcomes.

### 7.3. Sensitivity of the Methods to the Algorithm Setting

One of the main difficulties in using SA-based methods is related to the choice of the algorithm settings. This step could be particularly critical, especially for nonmodeller users. The problem is even worsened by the lack of a theoretically based criterion generally valid for all conditions and all the tracers. Empirical rules have been presented in literature, although their validity is case-dependent.

All the SA methods, standard and filtered versions, require the* a priori* definition of the basis function for the spectral components. The beta grid has to be designed to appropriately cover the distribution of all the kinetic components detectable from the PET data, making it denser where the probability of measuring a component is higher. Thus, the choice of beta grid can be seen as a way of applying* a priori* information on the quantification: the more accurate this information is, the better the quantification results are. It has to be noticed that the relationship between basis function and final results is not well-formulated as the Bayesian methods. In practice, logarithmic distributions represent the most common solutions, although it has been shown that other choices of *β* distribution, like equiangular or orthogonal basis, might improve the performance of SA methods, both standard and filtered versions [23, 30].

The number of grid components appears to be less critical than the choice of their distributions. Different attempts have been done comparing SA results with a different number of *β*'s [24, 97]: the advantages of a more populated grid, especially in terms of accuracy and precision of spectral estimates, do not counterbalance the relevant increase of computation time required by the algorithm. On the contrary, a smaller number of components could be critical if not optimally distributed around the true kinetic components. As suggested by Rizzo and colleagues [38], 20/30 elements are adequate only when accurate prior information is available. If this condition is not verified the final estimates can be strongly biased. Since the results obtained in the literature confirmed that a grid consisting of 100 *β*s represents a good trade-off between estimate precision and algorithm efficiency, we recommend it for both filtered and standard SA applications.

In respect to the standard SA, filtered SA methods require also the filtering definition. Spectral filtering heavily impacts on the final performance of the methods and thus it has to be carefully managed, depending on the peculiarities of SA algorithm used. For SAIF, for example, the choice of the filter passband coincides with the assumption of the kinetic interval within which the investigation processes can be detected. Outside this interval, all the kinetic components are considered as a mix of noise, random SA effects, and true kinetic components (like blood volume or trapping) and thus removed from the estimated spectrum. For SA filtering definition, different strategies are available depending on the particular dataset and tracer under analysis. The most common approach consists in the use of model-based simulations to generate a mixture of tissue TACs representing the tracer kinetics in the tissues of interest. Then, the best filter setting is defined as the one that minimizes the bias of the parameters of interest [21, 24]. Hierarchical approaches, from ROI level to voxel level, are also applied to set filtering parameters for parametric mapping [98, 99]. Independently from the strategy used for filter definition, it is always recommended to verify* a posteriori* the assumption correctness by assessing whether the distribution of estimated spectral components falls within the filter interval rather than accumulating at its extremes.

## 8. Conclusions

Quantification of dynamic PET studies can be performed in several different ways, which mainly differ in the assumptions about the system of interest and in the analysis outcomes. In this review we focused on spectral analysis, a general and flexible quantification method based on minimal model assumptions. Considering the trade-off between outcomes and requirements, spectral analysis can be located in an intermediate position between graphical methods and compartmental modelling.

In its original formulation, the main limitation of ESA is related to its sensitivity to the noise in the data. For this reason, filtered solutions have been proposed, with the most prominent nowadays being RS and SAIF. Literature results have shown that SA-based approaches are robust and reliable quantification methods, applicable for both region and voxel-wise analysis (especially as regards filtered versions), in different tracers, anatomical systems (brain and nonbrain), and physiological conditions.

## Figures and Tables

**Table 1:** Overview of Spectral Analysis methods.

Full Name	Exponential Spectral Analysis	Rank-shaping spectral analysis	Spectral analysis with iterative filter	Nonlinear spectral analysis
Description				
Short name	ESA	RS	SAIF	NLSA
Type of tracer kinetics	Any	Reversible tracers only	Irreversible tracers only	Any
Main parameters of interest	*V*_*T*_/*K*_*i*_, *V*_*b*_, *K*_1_	*V*_*T*_	*K*_*i*_, *V*_*b*_, *K*_1_	*V*_*T*_/*K*_*i*_, *V*_*b*_, *K*_1_
Required settings	Grid of components	Grid of components SNR	Grid of components Filter passband	None

Outputs				
Data fit	✓	✓	✓	✓
Kinetic spectrum	✓	—	✓	✓
ROI analysis	✓	—	✓	✓
VOXEL analysis	—	✓	✓	—

*V*_*T*_: distribution volume; *K*_*i*_: trapping rate constant; *V*_*b*_: blood volume; *K*_1_: weighted average influx rate constant; SNR: signal-to-noise ratio.

## References

[1] Bertoldo A., Rizzo G., Veronese M. (2014). Deriving physiological information from PET images: from SUV to compartmental modelling. *Clinical and Translational Imaging*.

[2] Kubota K. (1985). Lung tumor imaging by positron emission tomography using C-11 L-methionine. *Journal of Nuclear Medicine*.

[3] Kim C. K., Gupta N. C., Chandramouli B., Alavi A. (1994). Standardized uptake values of FDG: body surface area correction is preferable to body weight correction. *Journal of Nuclear Medicine*.

[4] Thie J. A. (2004). Understanding the standardized uptake value, its methods, and implications for usage. *Journal of Nuclear Medicine*.

[5] Boellaard R., Krak N. C., Hoekstra O. S., Lammertsma A. A. (2004). Effects of noise, image resolution, and ROI definition on the accuracy of standard uptake values: a simulation study. *Journal of Nuclear Medicine*.

[6] Hamberg L. M., Hunter G. J., Alpert N. M., Choi N. C., Babich J. W., Fischman A. J. (1994). The dose uptake ratio as an index of glucose metabolism: useful parameter or oversimplification?. *Journal of Nuclear Medicine*.

[7] Huang S.-C. (2000). Anatomy of SUV. *Nuclear Medicine and Biology*.

[8] Logan J., Fowler J. S., Volkow N. D. (1990). Graphical analysis of reversible radioligand binding from time-activity measurements applied to [N-11C-methyl]-(-)-cocaine PET studies in human subjects. *Journal of Cerebral Blood Flow & Metabolism*.

[9] Patlak C. S., Blasberg R. G., Fenstermacher J. D. (1983). Graphical evaluation of blood-to-brain transfer constants from multiple-time uptake data. *Journal of Cerebral Blood Flow and Metabolism*.

[10] Slifstein M., Laruelle M. (2001). Models and methods for derivation of in vivo neuroreceptor parameters with PET and SPECT reversible radiotracers. *Nuclear Medicine and Biology*.

[11] Godfrey K. (1982). *Compartmental Models and Their Application*.

[12] Phelps M. E. (2004). *PET: Molecular Imaging and Its Biological Applications*.

[13] Gunn R. N., Gunn S. R., Cunningham V. J. (2001). Positron emission tomography compartmental models. *Journal of Cerebral Blood Flow and Metabolism*.

[14] Cunningham V. J., Jones T. (1993). Spectral analysis of dynamic PET studies. *Journal of Cerebral Blood Flow & Metabolism*.

[15] Schmidt K. (1999). Which linear compartmental systems can be analyzed by spectral analysis of PET output data summed over all compartments?. *Journal of Cerebral Blood Flow and Metabolism*.

[16] Cobelli C., Carson E. (2008). *Introduction to Modeling in Physiology and Medicine*.

[17] Cobelli C., Foster D., Toffolo G. (2000). *Tracer Kinetics in Biomedical Research*.

[18] Carson R. E. (2005). Tracer kinetic modeling in PET. *Positron Emission Tomography*.

[19] Ichise M., Meyer J. H., Yonekura Y. (2001). An introduction to PET and SPECT neuroreceptor quantification models. *Journal of Nuclear Medicine*.

[20] Innis R. B., Cunningham V. J., Delforge J. (2007). Consensus nomenclature for *in vivo* imaging of reversibly binding radioligands. *Journal of Cerebral Blood Flow & Metabolism*.

[21] Turkheimer F., Moresco R. M., Lucignani G., Sokoloff L., Fazio F., Schmidt K. (1994). The use of spectral analysis to determine regional cerebral glucose utilization with positron emission tomography and [_18_F]fluorodeoxyglucose: theory, implementation, and optimization procedures. *Journal of Cerebral Blood Flow & Metabolism*.

[22] DiStefano J. J. (1981). Optimized blood sampling protocols and sequential design of kinetic experiments. *American Journal of Physiology—Regulatory Integrative and Comparative Physiology*.

[23] Cunningham V. J., Gunn R. N., Byrne H., Matthews J. C. (1998). Suppression of noise artifacts in spectral analysis of dynamic PET data. *Quantitative Functional Brain Imaging with Positron Emission Tomography*.

[24] Veronese M., Bertoldo A., Bishu S. (2010). A spectral analysis approach for determination of regional rates of cerebral protein synthesis with the L-[1-(11)C]leucine PET method. *Journal of Cerebral Blood Flow & Metabolism*.

[25] Lewicki M. S., Sejnowski T. J. (2000). Learning overcomplete representations. *Neural Computation*.

[26] Schmidt K. C., Turkheimer F. E. (2002). Kinetic modeling in positron emission tomography. *Quarterly Journal of Nuclear Medicine*.

[27] Turkheimer F., Sokoloff L., Bertoldo A. (1998). Estimation of component and parameter distributions in spectral analysis. *Journal of Cerebral Blood Flow and Metabolism*.

[28] Lammertsma A. A., Hume S. P. (1996). Simplified reference tissue model for PET receptor studies. *NeuroImage*.

[29] Maltz J. S. (2002). Parsimonious basis selection in exponential spectral analysis. *Physics in Medicine and Biology*.

[30] Turkheimer F. E., Hinz R., Gunn R. N., Aston J. A. D., Gunn S. R., Cunningham V. J. (2003). Rank-shaping regularization of exponential spectral analysis for application to functional parametric mapping. *Physics in Medicine and Biology*.

[31] Gunn R. N., Gunn S. R., Turkheimer F. E., Aston J. A. D., Cunningham V. J. (2002). Positron emission tomography compartmental models: a basis pursuit strategy for kinetic modeling. *Journal of Cerebral Blood Flow and Metabolism*.

[32] Peng J.-Y., Aston J. A. D., Gunn R. N., Liou C.-Y., Ashburner J. (2008). Dynamic positron emission tomography data-driven analysis using sparse Bayesian learning. *IEEE Transactions on Medical Imaging*.

[33] Takodoro M., Jones A. K. P., Cunningham V. J. (1993). Parametric images of C-11-diprenorphine binding using spectral analysis of dynamic PET images acquired in 3D. *Quantification of Brain Function: Tracer Kinetics and Image Analysis in Brain PET*.

[34] Weeks R. A., Cunningham V. J., Piccini P., Waters S., Harding A. E., Brooks D. J. (1997). 11C-diprenorphine binding in Huntington's disease: a comparison of region of interest analysis with statistical parametric mapping. *Journal of Cerebral Blood Flow and Metabolism*.

[35] Turkheimer F. E., Banati R. B., Visvikis D., Aston J. A. D., Gunn R. N., Cunningham V. J. (2000). Modeling dynamic PET-SPECT studies in the wavelet domain. *Journal of Cerebral Blood Flow and Metabolism*.

[36] Turkheimer F. E., Brett M., Visvikis D., Cunningham V. J. (1999). Multiresolution analysis of emission tomography images in the wavelet domain. *Journal of Cerebral Blood Flow and Metabolism*.

[37] Jiang C.-R., Aston J. A. D., Wang J.-L. (2009). Smoothing dynamic positron emission tomography time courses using functional principal components. *NeuroImage*.

[38] Rizzo G., Turkheimer F. E., Keihaninejad S., Bose S. K., Hammers A., Bertoldo A. (2012). Multi-scale hierarchical generation of PET parametric maps: application and testing on a [^11^C]DPN study. *NeuroImage*.

[39] Huang S.-C., Zhou Y., Stout D., Barrio J. R. Image-wise model fitting for generating parametric images in dynamic PET studies.

[40] Huang S.-C., Zhou Y. (1998). Spatially-coordinated regression for image-wise model fitting to dynamic pet data for generating parametric images. *IEEE Transactions on Nuclear Science*.

[41] Zhou Y., Huang S.-C., Bergsneider M., Wong D. F. (2002). Improved parametric image generation using spatial-temporal analysis of dynamic PET studies. *NeuroImage*.

[42] Veronese M., Schmidt K. C., Smith C. B., Bertoldo A. (2012). Use of spectral analysis with iterative filter for voxelwise determination of regional rates of cerebral protein synthesis with L-[1- 11C]leucine PET. *Journal of Cerebral Blood Flow and Metabolism*.

[43] Rizzo G., Veronese M., Zanotti-Fregonara P., Bertoldo A. (2013). Voxelwise quantification of [_11_C](*R*)-rolipram PET data: a comparison between model-based and data-driven methods. *Journal of Cerebral Blood Flow & Metabolism*.

[44] Veronese M., Rizzo G., Turkheimer F. E., Bertoldo A. (2013). SAKE: a new quantification tool for positron emission tomography studies. *Computer Methods and Programs in Biomedicine*.

[45] Meikle S. R., Matthews J. C., Cunningham V. J. (1998). Parametric image reconstruction using spectral analysis of PET projection data. *Physics in Medicine and Biology*.

[46] Tsoumpas C., Turkheimer F. E., Thielemans K. (2008). A survey of approaches for direct parametric image reconstruction in emission tomography. *Medical Physics*.

[47] Bertoldo A., Peltoniemi P., Oikonen V., Knuuti J., Nuutila P., Cobelli C. (2001). Kinetic modeling of [^18^F]FDG in skeletal muscle by PET: A four-compartment five-rate-constant model. *American Journal of Physiology—Endocrinology and Metabolism*.

[48] Bertoldo A., Price J., Mathis C. (2005). Quantitative assessment of glucose transport in human skeletal muscle: dynamic positron emission tomography imaging of [O-methyl-^11^C]3-O-methyl-D-glucose. *The Journal of Clinical Endocrinology & Metabolism*.

[49] Bertoldo A., Vicini P., Sambuceti G., Lammertsma A. A., Parodi O., Cobelli C. (1998). Evaluation of compartmental and spectral analysis models of [18F]FDG kinetics for heart and brain studies with PET. *IEEE Transactions on Biomedical Engineering*.

[50] Myers J. F. M., Rosso L., Watson B. J. (2012). Characterisation of the contribution of the GABA-benzodiazepine *α*1 receptor subtype to [^11^C]Ro15-4513 PET images. *Journal of Cerebral Blood Flow & Metabolism*.

[51] Grecchi E., Veronese M., Moresco R. M. (2016). Quantification of dynamic [18F]FDG pet studies in acute lung injury. *Molecular Imaging and Biology*.

[52] Mariotti E., Veronese M., Dunn J. T., Southworth R., Eykyn T. R. (2015). Kinetic analysis of hyperpolarized data with minimum a priori knowledge: hybrid maximum entropy and nonlinear least squares method (MEM/NLS). *Magnetic Resonance in Medicine*.

[53] Di Bella E. V., Sitek A. Time curve analysis techniques for dynamic contrast MRI studies.

[54] Liberati D., Turkheimer F. (1999). Linear spectral deconvolution of catabolic plasma concentration decay in dialysis. *Medical and Biological Engineering and Computing*.

[55] Salat D. H., Buckner R. L., Snyder A. Z. (2004). Thinning of the cerebral cortex in aging. *Cerebral Cortex*.

[56] Herscovitch P., Raichle M. E. (1983). Effect of tissue heterogeneity on the measurement of cerebral blood flow with the equilibrium C^15^O_2_ inhalation technique. *Journal of Cerebral Blood Flow & Metabolism*.

[57] Herholz K., Patlak C. S. (1987). The influence of tissue heterogeneity on results of fitting nonlinear model equations to regional tracer uptake curves: with an application to compartmental models used in positron emission tomography. *Journal of Cerebral Blood Flow and Metabolism*.

[58] Blomqvist G., Lammertsma A. A., Mazoyer B., Wienhard K. (1995). Effect of tissue heterogeneity on quantification in positron emission tomography. *European Journal of Nuclear Medicine*.

[59] Veronese M., Rizzo G., Aboagye E. O., Bertoldo A. (2014). Parametric imaging of 18F-fluoro-3-deoxy-3-L-fluorothymidine PET data to investigate tumour heterogeneity. *European Journal of Nuclear Medicine and Molecular Imaging*.

[60] Zhou Y., Huang S. C., Cloughesy T., Hoh C. K., Black K., Phelps M. E. (1997). A modeling-based factor extraction method for determining spatial heterogeneity of Ga-68 EDTA kinetics in brain tumors. *IEEE Transactions on Nuclear Science*.

[61] Bentourkia M. (2003). PET kinetic modeling of 11C-acetate from projections. *Computerized Medical Imaging and Graphics*.

[62] Marshall R. C., Powers-Risius P., Reutter B. W. (2004). Kinetic analysis of 18F-fluorodihydrorotenone as a deposited myocardial flow tracer: comparison to 201Tl. *Journal of Nuclear Medicine*.

[63] Green L. A., Nguyen K., Berenji B. (2004). A tracer kinetic model for 18F-FHBG for quantitating herpes simplex virus type 1 thymidine kinase reporter gene expression in living animals using PET. *Journal of Nuclear Medicine*.

[64] Meikle S. R., Eberl S., Iida H. (2001). Instrumentation and methodology for quantitative pre-clinical imaging studies. *Current Pharmaceutical Design*.

[65] Hammers A., Asselin M.-C., Turkheimer F. E. (2007). Balancing bias, reliability, noise properties and the need for parametric maps in quantitative ligand PET: [^11^C]diprenorphine test-retest data. *NeuroImage*.

[66] Price J. C., Klunk W. E., Lopresti B. J. (2005). Kinetic modeling of amyloid binding in humans using PET imaging and Pittsburgh Compound-B. *Journal of Cerebral Blood Flow and Metabolism*.

[67] Miederer I., Ziegler S. I., Liedtke C. (2009). Kinetic modelling of [11C]flumazenil using data-driven methods. *European Journal of Nuclear Medicine and Molecular Imaging*.

[68] Zanotti-Fregonara P., Leroy C., Roumenov D., Trichard C., Martinot J.-L., Bottlaender M. (2013). Kinetic analysis of [11C]befloxatone in the human brain, a selective radioligand to image monoamine oxidase A. *EJNMMI Research*.

[69] Zanotti-Fregonara P., Xu R., Zoghbi S. S. (2016). The PET radioligand ^18^F-FIMX images and quantifies metabotropic glutamate receptor 1 in proportion to the regional density of its gene transcript in human brain. *Journal of Nuclear Medicine*.

[70] Bertoldo A., Vicini P., Sambuceti G., Lammertsma A. A., Parodi O., Cobelli C. (1998). Evaluation of compartmental and spectral analysis models of [^18^F]FDG kinetics for heart and brain studies with PET. *IEEE Transactions on Biomedical Engineering*.

[71] Gullberg G. T., Reutter B. W., Sitek A., Maltz J. S., Budinger T. F. (2010). Dynamic single photon emission computed tomography—basic principles and cardiac applications. *Physics in Medicine and Biology*.

[72] Pencek R. R., Bertoldo A., Price J., Kelley C., Cobelli C., Kelley D. E. (2006). Dose-responsive insulin regulation of glucose transport in human skeletal muscle. *American Journal of Physiology - Endocrinology and Metabolism*.

[73] Gheysens O., Postnov A., Deroose C. M. (2015). Quantification, variability, and reproducibility of basal skeletal muscle glucose uptake in healthy humans using ^18^F-FDG PET/CT. *Journal of Nuclear Medicine*.

[74] Puri T., Blake G. M., Frost M. L. (2012). Comparison of six quantitative methods for the measurement of bone turnover at the hip and lumbar spine using 18F-fluoride PET-CT. *Nuclear Medicine Communications*.

[75] Siddique M., Frost M. L., Blake G. M. (2011). The precision and sensitivity of 18F-fluoride PET for measuring regional bone metabolism: a comparison of quantification methods. *Journal of Nuclear Medicine*.

[76] Murase K., Tsuda T., Mochizuki T., Ikezoe J. (1998). A simplified method for the quantitative analysis of 99Tcm-GSA liver scintigraphy using spectral analysis. *Nuclear Medicine Communications*.

[77] Murase K., Tsuda T., Mochizuki T., Ikezoe J. (1999). Hepatic extraction fraction of hepatobiliary radiopharmaceuticals measured using spectral analysis. *Nuclear Medicine Communications*.

[78] Murase K., Yamazaki Y., Mochizuki T., Ikezoe J. (2002). Renal uptake rate measurement of ^99m^Tc-dimercaptosuccinic acid using spectral analysis. *Nuclear Medicine Communications*.

[79] Tomasi G., Kenny L., Mauri F., Turkheimer F., Aboagye E. O. (2011). Quantification of receptor-ligand binding with [^18^F]fluciclatide in metastatic breast cancer patients. *European Journal of Nuclear Medicine and Molecular Imaging*.

[80] Tomasi G., Turkheimer F., Aboagye E. (2012). Importance of quantification for the analysis of PET data in oncology: review of current methods and trends for the future. *Molecular Imaging and Biology*.

[81] Kenny L. M., Vigushin D. M., Al-Nahhas A. (2005). Quantification of cellular proliferation in tumor and normal tissues of patients with breast cancer by [^18^F]fluorothymidine-positron emission tomography imaging: evaluation of analytical methods. *Cancer Research*.

[82] Wells P., Aboagye E., Gunn R. N. (2003). 2-[^11^C]thymidine positron emission tomography as an indicator of thymidylate synthase inhibition in patients treated with AG337. *Journal of the National Cancer Institute*.

[83] Verwer E. E., Bahce I., Van Velden F. H. P. (2014). Parametric methods for quantification of 18F-FAZA kinetics in non-small cell lung cancer patients. *Journal of Nuclear Medicine*.

[84] Schmidvolker V. (2010). Kinetic models for cancer imaging. *Advances in Computational Biology*.

[85] Turkheimer F. E., Edison P., Pavese N. (2007). Reference and target region modeling of [11C]-(R)-PK11195 brain studies. *Journal of Nuclear Medicine*.

[86] Turkheimer F. E., Selvaraj S., Hinz R. (2012). Quantification of ligand PET studies using a reference region with a displaceable fraction: application to occupancy studies with [^11^C]-DASB as an example. *Journal of Cerebral Blood Flow & Metabolism*.

[87] Riaño Barros D. A., McGinnity C. J., Rosso L. (2014). Test-retest reproducibility of cannabinoid-receptor type 1 availability quantified with the PET ligand [^11^C]MePPEP. *NeuroImage*.

[88] McGinnity C. J., Hammers A., Barros D. A. R. (2014). Initial evaluation of ^18^F-GE-179, a putative PET tracer for activated N-methyl D-aspartate receptors. *Journal of Nuclear Medicine*.

[89] Salinas C. A., Searle G. E., Gunn R. N. (2015). The simplified reference tissue model: model assumption violations and their impact on binding potential. *Journal of Cerebral Blood Flow & Metabolism*.

[90] Galazzo I. B., Bose S. K., Ramlackhansingh A. F. (2010). Kinetic modeling of the adenosine A2A subtype receptor radioligand [11C]SCH442416 in humans. *NeuroImage*.

[91] Rizzo G., Veronese M., Tonietto M., Zanotti-Fregonara P., Turkheimer F. E., Bertoldo A. (2014). Kinetic modeling without accounting for the vascular component impairs the quantification of [^11^C]PBR28 brain PET data. *Journal of Cerebral Blood Flow & Metabolism*.

[92] Tomasi G., Kimberley S., Rosso L., Aboagye E., Turkheimer F. (2012). Double-input compartmental modeling and spectral analysis for the quantification of positron emission tomography data in oncology. *Physics in Medicine and Biology*.

[93] Chen K., Bandy D., Reiman E. (1998). Noninvasive quantification of the cerebral metabolic rate for glucose using positron emission tomography, 18F-fluoro-2-deoxyglucose, the Patlak method, and an image-derived input function. *Journal of Cerebral Blood Flow and Metabolism*.

[94] Vriens D., De Geus-Oei L.-F., Oyen W. J. G., Visser E. P. (2009). A curve-fitting approach to estimate the arterial plasma input function for the assessment of glucose metabolic rate and response to treatment. *Journal of Nuclear Medicine*.

[95] Syvänen S., Blomquist G., Appel L., Hammarlund-Udenaes M., Långström B., Bergström M. (2006). Predicting brain concentrations of drug using positron emission tomography and venous input: modeling of arterial-venous concentration differences. *European Journal of Clinical Pharmacology*.

[96] Tonietto M., Rizzo G., Veronese M., Bertoldo A. Modelling arterial input functions in positron emission tomography dynamic studies.

[97] Tomasi G., Bertoldo A., Bishu S., Unterman A., Smith C. B., Schmidt K. C. (2009). Voxel-based estimation of kinetic model parameters of the L-1- 11 Cleucine PET method for determination of regional rates of cerebral protein synthesis: Validation and comparison with region-of-interest-based methods. *Journal of Cerebral Blood Flow and Metabolism*.

[98] Rizzo G., Turkheimer F. E., Bertoldo A. (2013). Multi-scale hierarchical approach for parametric mapping: assessment on multi-compartmental models. *NeuroImage*.

[99] Zanderigo F., Ogden R. T., Bertoldo A., Cobelli C., Mann J. J., Parsey R. V. (2010). Empirical Bayesian estimation in graphical analysis: a voxel-based approach for the determination of the volume of distribution in PET studies. *Nuclear Medicine and Biology*.

